# Why don’t humanitarian organizations provide safe abortion services?

**DOI:** 10.1186/s13031-016-0075-8

**Published:** 2016-03-24

**Authors:** Therese McGinn, Sara E. Casey

**Affiliations:** Heilbrunn Department of Population and Family Health, Mailman School of Public Health, Columbia University, 60 Haven Ave, New York, NY 10032 USA

**Keywords:** Unsafe abortion, Abortion-related maternal mortality, Humanitarian settings, Sexual violence

## Abstract

**Background:**

Although sexual and reproductive health services have become more available in humanitarian settings over the last decade, safe abortion services are still rarely provided. The authors’ observations suggest that four reasons are typically given for this gap: ‘There’s no need’; ‘Abortion is too complicated to provide in crises’; ‘Donors don’t fund abortion services’; and ‘Abortion is illegal’.

**Discussion:**

However, each of these reasons is based on false premises. Unsafe abortion is a major cause of maternal mortality globally, and the collapse of health systems in crises suggests it likely increases in humanitarian settings. Abortion procedures can be safely performed in health centers by mid-level providers without sophisticated equipment or supplies. Although US government aid does not fund abortion-related activities, other donors, including many European governments, do fund abortion services. In most countries, covering 99 % of the world’s population, abortion is permitted under some circumstances; it is illegal without exception in only six countries. International law supports improved access to safe abortion.

**Summary:**

As none of the reasons often cited for not providing these services is valid, it is the responsibility of humanitarian NGOs to decide where they stand regarding their commitment to humanitarian standards and women’s right to high quality and non-discriminatory health services. Providing safe abortion to women who become pregnant as a result of rape in war may be a more comfortable place for organizations to begin the discussion. Making safe abortion available will improve women’s health and human rights and save lives.

## Background

In the early 1990s, the sexual and reproductive health (SRH) needs of people affected by conflict or natural disaster were rarely met. A 1993 editorial in The Lancet identified SRH services as a complete gap in refugee settings [1]. The groundbreaking 1994 report, *Refugee Women and Reproductive Health Care: Reassessing Priorities*, described how the health of women fleeing war or natural disasters was further threatened by the near complete absence of SRH services [2]. Refugee women spoke about their SRH needs at the 1994 International Conference on Population and Development in Cairo [3]. Also at this time, extensive media attention to the plight of women in the Bosnia and Rwanda crises raised awareness of SRH, especially sexual violence, in crises. This spurred international attention to the issue and led to the development of coalitions such as the Inter-agency Working Group on Reproductive Health in Crises (IAWG) and the Reproductive Health Response in Conflict Consortium; these groups in turn led to development of policy, technical and program guides, including a 1999 comprehensive field guide, updated in 2010 as the Inter-agency field manual on reproductive health in humanitarian settings [4]. Despite progress, UNFPA’s 2015 State of World Population documented the growing SRH needs in emergencies and called for increased global commitment to meet them [5].

In the 20 years since *Reassessing Priorities* was published, access to SRH services in humanitarian settings has improved. A minimum set of priority SRH activities to implement in the earliest days of a humanitarian crisis, the Minimum Initial Services Package (MISP) for SRH, was added in 2004 in the Sphere Project’s Humanitarian Charter and Minimum Standards in Disaster Response [6]. In 2004, the IAWG implemented a global evaluation of SRH in humanitarian settings which showed that some elements of SRH, such as antenatal care, short-acting contraceptive methods or HIV prevention, were generally available, while others, like post-abortion care, long-acting and permanent contraceptive methods, care for survivors of gender-based violence and HIV care and treatment were less often available [7]. Safe abortion services were not available and were barely addressed in the final report. A second global evaluation completed in 2014 showed improvement in services for HIV and gender-based violence but not in the availability of safe abortion or the full range of contraceptive services that could preclude some of the need for safe abortion [8, 9]. Nor was abortion mentioned, except for rare references to post-abortion care, in an analysis of health and protection proposals [10].

Although unintended pregnancy is a problem everywhere and can be a special concern for women affected by humanitarian emergencies [4, 11], data on the extent of the need for safe abortion in humanitarian settings are lacking. A literature search in Medline and Pubmed identified only two articles published on induced abortion in humanitarian settings in the last 10 years: one in 2007 described the unsafe abortion techniques used by Burmese refugee women in Thailand [12] and the other in 2002 called on the international community to provide safe abortion for refugees [11]. The IAWG recognized the gap and added a chapter on comprehensive abortion care to the 2010 revision of the Inter-agency field manual on reproductive health in humanitarian settings [4]. Aside from this small advance, little has changed: safe abortion services are rarely available to women affected by war or natural disasters. Why don’t humanitarian organizations provide safe abortion in humanitarian settings? Based on numerous discussions and observations by the authors over many years, four reasons are typically articulated to account for this: ‘There’s no need;’ ‘Abortion is too complicated to provide in crises;’ ‘Donors don’t fund abortion services;’ and ‘Abortion is illegal.’ We will consider the evidence for each of these assertions in turn.

## Discussion

### There’s no need

The lack of reliable data published on unsafe abortion in humanitarian settings must not be interpreted to mean there is no need for safe abortion services. Unsafe abortion is defined by the World Health Organization (WHO) as ‘a procedure for terminating an unintended pregnancy carried out either by persons lacking the necessary skills or in an environment that does not conform to minimal medical standards, or both’ [13]. Reliable data on unsafe abortion overall are lacking [14], and this gap is even more pronounced in humanitarian settings. Analysis of 2008 data found that 21 % of pregnancies globally end in abortion, and the vast majority (86 %) occur in developing countries [15]. Nearly half of abortions worldwide, and 97 % of those in sub-Saharan Africa, are unsafe [15]. Up to 50 % of women who have unsafe abortions seek care for complications, including hemorrhage, sepsis, perforated uterus and trauma to internal organs [14]. The risk of death due to unsafe abortion is 90 unsafe abortion-related maternal deaths per 100,000 live births in sub-Saharan Africa, three times the global average of 30 per 100,000 live births [16]. Data are even more scarce regarding the scope of the long term health consequences of unsafe abortion which include chronic infections and infertility [14]. However, a recent systematic review found a ratio of 596 severe complications due to abortion per 100,000 live births (range 435–5298), much greater than that for abortion-related deaths [17].

These global data indicate that unsafe abortion exists in many countries and, given the nature of humanitarian emergencies, the need for safe abortion services likely increases in humanitarian settings. The collapse of health systems during a humanitarian emergency reduces access to health care for pregnant women, including emergency obstetric care and safe delivery services [18, 19]. Women who wish to delay pregnancy may have little access to contraceptive services, and women who experience an unwanted pregnancy are unlikely to have access to safe abortion services, perhaps leading them to seek an unsafe abortion [19]. In addition, sexual violence has long been associated with war and has been documented in numerous humanitarian settings [20–24]. Survivors of rape experience negative physical, psychological and social outcomes that may be further exacerbated when the rape results in pregnancy [22]. Unintended pregnancies and therefore unsafe abortions happen everywhere and are a major cause of maternal mortality, so making safe abortion available is critical to saving women’s lives.

### Abortion is too complicated to provide in crises

Another reason often cited for not providing safe abortion services is that they are ‘too complicated’ to provide in humanitarian settings. However, the protocol for safe abortion is well established, with manual vacuum aspiration (MVA) and medication abortion the recommended methods for first trimester abortion, when the vast majority of abortions are performed [25, 26]. Abortion procedures are among the safest medical procedures, with very low risk of morbidity and mortality [14, 25, 26]. Both MVA and medication abortion can be safely performed in the first trimester by trained mid-level providers [25, 27, 28]; a 2015 WHO guideline further described the technical evidence for abortion provision by mid-level providers [29], a position endorsed in a Lancet editorial [30]. Neither method requires electricity, running water or sophisticated equipment. Abortion can be safely provided in primary health care facilities (e.g., in health centers) [25]. Abortion with MVA takes 3–10 min to complete and most women can leave the facility after 30 min of post-procedure observation [25]. Most of the equipment, medications and infection prevention procedures needed for safe abortion services are the same as those needed for basic emergency obstetric and other gynecology services. Basic emergency obstetric care, which includes the treatment of complications of abortion, is an established minimum standard of care to provide in humanitarian settings; a health facility that delivers basic emergency obstetric care has the capacity to provide safe abortion [4, 6]. Therefore, an NGO that supports primary health care services in humanitarian settings could provide safe abortion services will little additional input.

### Donors don’t fund abortion services

Another commonly cited reason for not providing safe abortion is that ‘donors don’t fund abortion services.’ The US government, a major donor of humanitarian aid, does indeed not fund abortion services. The Helms amendment to the 1973 Foreign Assistance Act prohibits US government (USG) funds from being used for the ‘performance of abortions as a method of family planning or to motivate or coerce any person to practice abortions’ [31], and applies to both American and foreign NGOs that receive USG funding. In 1994, the US Congress added the Leahy Amendment to clarify that ‘the term “motivate”, as it relates to family planning assistance, shall not be construed to prohibit the provision, consistent with local law, of information or counseling about all pregnancy options including abortion’ [32]. This permits NGOs to provide information or counseling on legal abortion services.

In 1984, President Reagan imposed the Mexico City policy, known as the ‘Global Gag rule,’ in an executive order which expanded the restrictions in the Helms Amendment. Since its introduction, this policy has been rescinded by every Democratic president and reinstated by every Republican president in the first days of their administrations. The Mexico City policy withheld USAID family planning funding from foreign NGOs that used any funds, including those received from non-USG sources, to provide abortion-related counseling, services or referrals or advocate for liberalization of local abortion laws. The policy made an exception to save the life of the woman and in cases of rape or incest. The Global Gag rule forced foreign NGOs to make a difficult decision – forgo USG funding or limit their activities to those deemed acceptable by the US government [33]. Neither the Helms amendment nor the Mexico City policy places any restrictions on providing post-abortion care, the treatment of complications of abortion. President Obama revoked the policy when he took office in 2009 so it is not currently in effect [34].

However, the US government is not the only donor of humanitarian aid; other donors do fund safe abortion services, including private foundations and other bilateral, particularly European, donors [14]. For example, the United Kingdom Department for International Development explicitly affirms their support to make safe abortion more accessible where permitted by law in order to reduce death or disability from unsafe abortion [35].

The USG policies on aid regarding abortion are opaque and confusing. This leads NGOs to restrict information on abortion, including counseling and referrals for women with unintended pregnancies, even more than is required by the actual laws [36, 37]. For example, the Helms Amendment does not prevent organizations from providing safe abortion with non-USG funding. The Leahy amendment indicates that organizations receiving USG funding may provide information on all pregnancy options, including abortion as consistent with local laws, during contraceptive consultations funded by USG. And the Mexico City policy, when it was in effect, applied only to foreign NGOs, not to US NGOs. Although the Helms amendment does not prohibit NGOs from using other funds to provide abortion, the administrative burden of separating USG funds from other donor funds may prevent them from implementing abortion-related activities even with those other funds. Given the multiplicity of rules, it is difficult for organizations to track which and when restrictions apply. The least confusing and ‘safest’ course of action for an agency managing many programs may appear to be a blanket prohibition on all abortion-related activities.

Some self-censorship may be due to NGOs’ fear that their other non-family planning USG funding might be reduced or cut altogether if they implement abortion-related activities. This may be an important consideration, but when the George W Bush administration proposed extending the Global Gag rule to the US’s global HIV/AIDS funding in 2003, its major partners in the initiative to prevent mother-to-child transmission of HIV refused to enforce the rule if it were applied to their funding. Ultimately, the administration backed down and never extended the rule to HIV funding [38], a demonstration that fear of losing funds need not result in inaction.

NGOs must be true to their missions and limit risk. Safe abortion as well as other SRH services may be perceived as easier to dismiss in the face of controversy and stigma that often surrounds women who seek abortions and health workers who provide them. Program managers are too often uninformed and unsure of what is permitted by law and policy, which makes it easier to neither discuss nor provide abortion services. The anxiety of contravening poorly understood US restrictions prevents many organizations from even discussing abortion out of concern that they may put their other USG funding at risk. It is important for NGOs to understand the laws and actual restrictions rather than censor themselves beyond what is required.

Some donors fund safe abortion services. Currently, humanitarian organizations may provide safe abortion services, as permitted by international or national law, to women affected by crises with non-USG funds, even if they also receive USG funding. They may also, with USG funds, provide information and referral to clients on legal abortion services.

### Abortion is illegal

Many NGOs assume that abortion is illegal and therefore cannot be provided in countries where they work. In fact, this is rarely true. Abortion is not allowed under any circumstances in only six countries: Chile, Dominican Republic, El Salvador, Malta, Nicaragua and the Vatican [39]. In all other countries, covering 99 % of the world’s population, abortion is permitted under some circumstances. As of 2013, of 196 UN member and non-member states, 190 permitted abortion to save a woman’s life; 132 to preserve her health; 126 to preserve her mental health; 99 in cases of rape or incest, 69 for social or economic reasons and 58 permitted abortion on request [40]. The allowance for mental or physical health has been interpreted to permit abortion for rape or incest in countries without an explicit exemption for those situations. Three-quarters of the world’s population live in countries that permit abortion to preserve a woman’s mental health [39]. With a few exceptions, over time, countries have been liberalizing their abortion laws [40]. Among countries experiencing humanitarian crises or hosting displaced populations, Burkina Faso, Chad, Colombia, Ethiopia, Kenya, Mali and Sierra Leone have all liberalized their abortion laws since 1996 [39, 41].

In addition to national law, numerous instruments of international human rights and humanitarian law support improved access to safe abortion [14, 42]. The 179 country signatories of the 1994 Programme of Action of the International Conference on Population and Development (ICPD) committed to addressing the consequences of unsafe abortion and supported women’s right to SRH services [3]. The 1995 Beijing Platform for Action reinforced the ICPD Programme of Action and reaffirmed governments’ commitment to review punitive laws regarding abortion [43]. In Africa, the adoption of the Protocol to the African charter on human and people's rights on the rights of women in Africa (Maputo Protocol) in 2003 recognized the rights of women in Africa and explicitly endorsed access to comprehensive SRH care in Article 14, including access to safe abortion ‘in cases of sexual assault, rape, incest, and where the continued pregnancy endangers the mental and physical health of the mother or the life of the mother or the foetus’ [44]. Currently, 37 of 54 African Union member states have ratified the protocol, making it binding while another 14 countries have signed but not yet ratified it.

Restricting access to abortion does not decrease abortion rates. For example, Europe, which has relatively liberal abortion laws, has an abortion rate of 27 abortions per 1000 women aged 15–44 years while in eastern and middle Africa, where abortion is much more restricted, the rates are 38 and 36 per 1000 [15]. Further, unsafe abortion rates are more than four times higher in countries with restrictive abortion policies (26.7 unsafe abortions per 1000 women aged 15–44 years) than in countries with liberal policies (6.1 per 1000) [39].

Changes in abortion laws have been linked to changes in maternal mortality: when laws were made more restrictive, maternal mortality due to unsafe abortion increased while the reverse was true when restrictions on abortion were eased. For example, Romania reversed the legal status of abortion in 1966 and introduced further restrictions on access in 1985. Maternal mortality in Romania from 1979 to 1989 was ten times higher than in any other European country, mostly due to abortion-related deaths [45]. After legalization of abortion in 1989, mortality decreased from 170 deaths per 100,000 live births in 1989 to 60 in 1992 [45]. South Africa made abortion available on request in 1997 which resulted in a decline of 91 % in deaths due to unsafe abortion between 1994 and 1998–2001 [46]. The proportion of maternal mortality caused by complications of abortion in Ethiopia decreased from 22 to 41 % before 2002 to 6 % in 2007–2008, following a liberalization of the country’s abortion laws in 2004 [47].

Almost all-190 of 196-countries in the world permit abortion under some circumstances and access to abortion is supported by international treaties and law. Restricting access to abortion not only fails to reduce abortion rates but also increases maternal mortality. It is important that humanitarian organizations educate themselves about the laws of the countries in which they work and make safe abortion available to the degree permitted by law.

### A potential context to overcome misunderstandings: women who become pregnant as a result of rape in war

It may be more acceptable for humanitarian organizations to find common ground by discussing the provision of safe abortion to women who become pregnant from rape in war. The United Nations (UN) has specifically recognized systematic rape as a crime against humanity [48]. International attention to the use of rape as a weapon of war in the Bosnia and Rwanda crises of the mid-1990s and systematic rape in the Democratic Republic of Congo have raised the issue of how best to serve survivors of rape. Humanitarian health standards include psychosocial and clinical services for survivors of sexual assault including the provision of emergency contraception (EC) to prevent pregnancy, post-exposure prophylaxis (PEP) to prevent HIV infection and antibiotics to prevent sexually transmitted infections, if the woman presents at a health facility within the requisite time delay (within 5 days to receive EC and within 72 h for PEP) [4, 6]. However, safe abortion services are rarely provided, even where they are permitted by law [11]. The stigma surrounding rape can be much worse when a woman becomes pregnant as a result of rape and must carry the pregnancy to term-she may be rejected by her husband and her community; if she is unmarried, she may be rejected by potential marriage partners; she may suffer long-term psychological consequences as well as physical consequences of pregnancy [22, 49]. For example, women in Congo who had children as a result of rape were much more likely to report being isolated or rejected by their community [50].

Many experts in international humanitarian law consider the denial of safe abortion to survivors of rape a violation of the Geneva Conventions and international human rights law [42, 51, 52]. In 2013, the UN Security Council issued two resolutions that addressed sexual assault in armed conflict, ‘noting the need for access to the full range of sexual and reproductive health services, including regarding pregnancies resulting from rape, without discrimination’ [53] and called on countries to provide ‘non-discriminatory and comprehensive health services, including sexual and reproductive health’ [54]. These resolutions are interpreted to mean that safe abortion services should be provided to survivors of rape in war. Both the UK and Norway governments have specifically affirmed that safe abortion should be a component of medical treatment for women raped in war, and that international humanitarian law rather than national abortion laws are the legal standard to follow when treating survivors of rape in war [55, 56].

The Global Justice Center’s August 12th Campaign and European governments have challenged the US to respect the international treaties it has signed and remove restrictions on US humanitarian aid funding that deny safe abortion services to survivors of rape. Norway questioned the Helms’ amendment’s restrictions on foreign aid during the 2011 Universal Periodic Review of the United States before the Human Rights Council [57]. Further, the European Union (EU) explicitly reminded its member states that EU humanitarian aid should be kept independent of US restrictions on humanitarian aid to ensure access to abortion for survivors of rape in war [58, 59]. These policy discussions may facilitate changes in humanitarian NGOs’ practice regarding safe abortion in crises. Providing abortion to women who become pregnant as a result of rape may be a more acceptable entry point for a more thorough discussion on safe abortion for all women.

## Conclusion

The need for safe abortion services to reduce maternal mortality is clear. These services can be provided in health centers by mid-level providers. Abortion is permitted under some circumstances in all but six countries around the world; restricting access to abortion services does not reduce abortion rates. Although the US government does not fund abortion-related activities, other donors, including many European governments, do fund abortion services. These governments, some NGOs, and voices within the US government [60], are increasing the pressure on the US to respect international treaties and end restrictions on US funding that place them in violation of international law.

Currently, humanitarian organizations may provide safe abortion services, as permitted by international or national law, to women affected by crises with non-USG funds even if they also receive USG funding. Rather than permit US restrictions on abortion to dictate organizational policy, organizations should, at a minimum, begin the discussion about why they do not provide safe abortion. When discussing abortion, it should not be referred to as ‘illegal;’ language should reflect that it is legally permitted in some circumstances in all but six countries.

Global leaders will meet at the first World Humanitarian Summit in Turkey in May 2016 [61]. It is their responsibility, and that of humanitarian NGOs, to decide where they stand regarding their commitment to humanitarian standards and women’s right to high quality and non-discriminatory health services. Women who have unwanted pregnancies during a time of crisis should have access to safe abortion services. Providing safe abortion to women who become pregnant as a result of rape in war may be a more comfortable place for organizations to begin the discussion of access to safe abortion for all women. Making safe abortion available will improve women’s health and human rights and save lives.

## Abbreviations

EC: emergency contraception
EU: European Union
IAWG: Inter-agency Working Group on Reproductive Health in Crises
ICPD: International Conference on Population and Development
MISP: minimum initial services package
MVA: manual vacuum aspiration
NGO: non-governmental organization
PEP: post-exposure prophylaxis
SRH: sexual and reproductive health
UN: United Nations
USG: US Government
WHO: World Health Organization
